# IBKA-MSM: A Novel Multimodal Fake News Detection Model Based on Improved Swarm Intelligence Optimization Algorithm, Loop-Verified Semantic Alignment and Confidence-Aware Fusion

**DOI:** 10.3390/biomimetics10110782

**Published:** 2025-11-17

**Authors:** Guangyu Mu, Jiaxiu Dai, Chengguo Li, Jiaxue Li

**Affiliations:** 1 School of Management Science and Information Engineering, Jilin University of Finance and Economics, Changchun 130117, China; guangyumu@jlufe.edu.cn (G.M.); 6241191013@s.jlufe.edu.cn (J.D.); 2 Changchun Hongyu Technology Co., Ltd., Changchun 130507, China; lichengguo@hykj111.com; 3 Institute of National Development and Security Studies, Jilin University, Changchun 130012, China

**Keywords:** multimodal fake news detection, Improved Black-Winged Kite Algorithm, semantic alignment, confidence-aware fusion, swarm intelligence

## Abstract

With the proliferation of social media platforms, misinformation has evolved toward more diverse modalities and complex cross-semantic correlations. Accurately detecting such content, particularly under conditions of semantic inconsistency and uneven modality dependency, remains a critical challenge. To address this issue, we propose a multimodal semantic representation framework named IBKA-MSM, which integrates swarm-intelligence-based optimization with deep neural modeling. The framework first employs an Improved Black-Winged Kite Algorithm (IBKA) for discriminative feature selection, incorporating adaptive step-size control, an elite-memory mechanism enhanced by opposition perturbation, Gaussian-based local exploitation, and population diversity regulation through reinitialization. In addition, a Modality-Generated Loop Verification (MGLV) mechanism is designed to enhance semantic alignment, and a Semantic Confidence Matrix with Modality-Coupled Interaction (SCM-MCI) is introduced to achieve adaptive multimodal fusion. Experimental results demonstrate that IBKA-MSM achieves an accuracy of 95.80%, outperforming mainstream hybrid models. The F1 score is improved by approximately 2.8% compared to PSO and by 1.6% compared to BKA, validating the robustness and strong capability of the proposed framework in maintaining multimodal semantic consistency for fake news detection.

## 1. Introduction

With the rapid growth of social media and intelligent information dissemination technologies, fake news has become a significant challenge for global cybersecurity and public opinion governance. It spreads quickly, is highly deceptive, and exerts a broad social impact. The wide circulation of misinformation subtly influences public perception and judgment, leading to biased opinions and emotional polarization. In severe cases, it can lead to social panic and collective unrest, posing a threat to public safety and social stability. Therefore, effectively identifying and limiting the spread of misinformation has become an essential research topic in information science and social computing. The primary objective of fake news detection is to model the semantic and dissemination patterns of news, thereby distinguishing between false information and genuine content, and providing technical support for online governance and risk prevention.

Early studies mainly focus on single-modal text analysis. Because textual data are easy to obtain and highly interpretable, traditional approaches rely on handcrafted features and shallow classifiers for binary discrimination. However, these methods are limited in capturing complex semantics and latent logical inconsistencies. With advances in deep learning and pre-trained language models [1], methods based on semantic representation learning have gradually replaced traditional feature engineering, significantly improving the ability to model contextual and semantic dependencies. Nevertheless, fake news often combines text and images to mislead readers. Single-modality textual modeling alone cannot reveal potential semantic–visual inconsistencies. Consequently, the research focus has shifted toward multimodal detection [2], which jointly explores textual and visual features to achieve cross-modal semantic alignment and consistency verification, extending fake-news detection from the linguistic level to visual–semantic association modeling [3].

In multimodal detection, fusion strategies have evolved from early feature concatenation to deep interactive modeling. Early approaches concatenate textual and visual features for joint prediction, which fail to capture complementary or contradictory relations across modalities. With the advancement of deep learning, attention mechanisms, and Transformer-based architectures have significantly improved cross-modal alignment and interaction. These models utilize multi-head attention to learn semantic correlations across modalities, thereby enhancing the detection of text–image inconsistencies and multimodal fake news [4]. However, current methods still struggle with modality heterogeneity and semantic uncertainty. Because text and images differ in information granularity, abstraction level, and semantic salience, fusion may introduce semantic bias or noise. Moreover, deep neural networks often fall into local optima in high-dimensional spaces, making it challenging to balance global exploration with fine-grained local modeling. Recent studies have shown that contrastive learning and optimal transport can mitigate misalignment to some extent. However, achieving robust global semantic alignment remains a challenge [5].

Meanwhile, metaheuristic swarm intelligence optimization algorithms have provided new perspectives for multimodal feature selection and structure optimization [6]. Algorithms such as the Black-Winged Kite Algorithm (BKA) [7], Sparrow Search Algorithm (SSA) [8], and Whale Optimization Algorithm (WOA) [9] simulate natural foraging or cooperative behaviors and demonstrate strong global search ability and convergence performance in feature compression and parameter optimization. Among them, the recently proposed BKA shows remarkable performance in multimodal feature selection and high-dimensional parameter tuning due to its fast convergence and strong global exploration. However, when handling complex, multimodal, and high-dimensional non-convex search spaces, the original BKA still faces challenges such as insufficient step-size adaptation, unstable population diversity, and poor balance between exploration and exploitation. These issues underscore the need for further methodological improvements to enhance the robustness and efficiency of the approach.

The significant contributions of this paper are summarized as follows:

(1) We propose an Improved Black-Winged Kite Algorithm (IBKA) for cross-modal feature generation and optimization. The algorithm introduces an adaptive step-size update strategy, elite memory with opposition-based disturbance, Gaussian-based local refinement, and population diversity monitoring with re-initialization, ensuring a dynamic balance between global exploration and local exploitation.
(2) We design a Modality Generation and Loop Verification mechanism (MGLV) that achieves cyclic cross-modal semantic validation through semantic reconstruction and consistency constraints. By reinforcing semantic coherence during the closed-loop generation–verification process, MGLV ensures the alignability and interpretability of representations within a shared semantic space, effectively mitigating semantic drift caused by cross-modal discrepancies.
(3) We construct a Semantic Confidence Matrix and Modality-Cross Interaction mechanism (SCM-MCI) for adaptive confidence modeling and deep semantic interaction across modalities. The SCM module dynamically evaluates modality reliability during fusion based on semantic confidence distribution and adjusts contribution weights accordingly. On this basis, the MCI module introduces a bidirectional semantic propagation strategy to enhance semantic complementarity and information consistency, enabling highly correlated and stable fused representations in the shared semantic space.
(4) All the proposed enhancement modules are incorporated into the IBKA-MSM framework and comprehensively evaluated on a multimodal fake news detection benchmark. The experimental findings confirm notable improvements across various performance indicators, demonstrating the effectiveness and robustness of the framework in semantic alignment, modality interaction, and cross-modal feature representation.

## 2. Related Work

### 2.1. Single-Modal Fake News Detection

Single-modal fake news detection focuses on identifying misinformation using feature representations from a single modality, such as text, images, or audio. Early efforts primarily concentrated on semantic analysis and sentiment recognition in textual content. With the rapid expansion of social media, researchers have begun exploring pragmatic deception cues embedded in language. Studies based on linguistic style models indicated that deceptive texts often exhibit greater subjectivity and emotional intensity, providing valuable insights for subsequent deep representation learning [10,11].

During the deep learning era, Convolutional Neural Networks (CNNs) and Recurrent Neural Networks (RNNs) became mainstream architectures. CNNs are suitable for capturing local features in short texts, while RNNs and their variants, such as LSTM and BiLSTM, effectively model long-range contextual dependencies [12]. These methods achieved significant improvements on standard datasets such as LIAR and FakeNewsNet, marking a shift toward deep learning–based single-modal detection.

Later, attention-based methods further enhanced the capabilities of semantic representation for detecting fake news. Hierarchical Attention Networks (HAN) extract key informative sentences through hierarchical attention, improving sensitivity to fine-grained semantic differences [13,14]. Meanwhile, the introduction of Transformer architectures fundamentally transformed text modeling paradigms. Pre-trained language models (PLMs), including BERT and RoBERTa, captured semantic and syntactic relationships through self-supervised learning, achieving substantial performance gains in fake news detection tasks [15].

However, relying solely on textual information remains insufficient. Studies have shown that fake news often misleads readers through exaggerated rhetoric or implicit factual distortion, making it difficult for single-modality semantic modeling to capture hidden event-level logic inconsistencies [16,17]. To alleviate this, knowledge-enhanced representation methods combine external facts or knowledge graphs with language models to improve factual consistency assessment [18,19].

Within the visual modality, early studies focused on statistical visual features for forgery detection. Recently, deep convolutional models have replaced handcrafted features. Networks such as ResNet and EfficientNet are widely used in image forensics tasks due to their capability to detect visual discontinuities indicative of manipulation [20]. Moreover, the Vision Transformer (ViT) and Swin Transformer architectures have demonstrated strong global perception capabilities for detecting image-based misinformation.

In recent years, robustness under cross-lingual and cross-domain conditions has gained attention. Domain adaptation strategies have been shown to reduce distribution shifts between source and target social media environments, while style transfer and data augmentation techniques significantly enhance performance in low-resource scenarios [21]. These methods emphasize semantic consistency and contextual interpretability, providing reliable support for subsequent multimodal fake news detection research.

### 2.2. Multimodal Fake News Detection

Compared with single-modal approaches, multimodal fake news detection emphasizes capturing and assessing cross-modal semantic consistency. Fake news on social media frequently appears as “authentic images + misleading text” or “manipulated images + truthful descriptions,” making it difficult for unimodal cues to characterize complete deception patterns. Studies have shown that aligning textual and visual representations in a shared semantic space helps reduce modality discrepancies and improves the recognition of semantic conflicts and contextual mismatches [22]. Such consistency modeling provides interpretable foundations for detecting visually supported misinformation and semantic deception.

To address modality imbalance issues, some studies adopt staged or gated fusion strategies, which mitigate modality dominance by performing intra-modal purification followed by gradual cross-modal interactions [23]. These approaches maintain the integrity of discriminative features in high-redundancy settings and improve robustness against noisy samples [24]. Meanwhile, knowledge-enhanced methods integrate external knowledge graphs into multimodal representations, constraining factual consistency through entity relationships in graph structures. This enhances interpretability by exposing semantic inconsistencies between textual and visual content.

Another research direction investigates event transfer and cross-domain robustness problems. Due to substantial variations in modality distribution across events, detection performance often degrades in unseen scenarios. Event-invariant learning and adversarial optimization have been applied to mitigate domain shifts in multimodal representations, enabling stable performance under event migration [25,26]. This contributes to cross-platform and cross-lingual generalization in future multimodal detection.

With the rise of self-supervised learning, contrastive learning frameworks have been widely explored for cross-modal consistency modeling. By constructing matched and mismatched image–text pairs, models learn underlying cross-modal correspondences during pretraining [27]. These methods maintain strong discriminative power even under limited annotations and demonstrate good generalization on noisy social media data. Further research performs fine-grained semantic similarity analysis, revealing abnormal alignment patterns characteristic of misinformation [28].

In the fusion stage, dynamic gating and contextual reasoning mechanisms adaptively assign weights based on modality reliability, alleviating bias from over-dominant unimodal features [29]. When combined with attention, this enables accurate identification of samples with “visually salient yet semantically deceptive” content, thus improving detection accuracy. Recent studies also explored multilingual and multicultural multimodal corpora, achieving cross-platform transfer through shared semantic alignment strategies [30].

Regarding data resources, large-scale multimodal benchmarks such as Fakeddit [31] provide hierarchical annotations, offering a unified standard for studying semantic consistency, fusion strategies, and cross-domain robustness. Newer approaches further incorporate large language models for multimodal retrieval and factual verification, showing strong potential under weak supervision [32]. Overall, the field is transitioning from feature-level fusion to semantic-level consistency modeling and optimization-driven fusion learning, laying a solid foundation for feature selection and gating optimization strategies.

## 3. Method

This paper presents a multimodal fake news detection method based on the Improved Black-Winged Kite Algorithm (IBKA), emphasizing the synergistic design of feature optimization and semantic fusion to enable efficient integration and robust discrimination of cross-modal information. To address the shortcomings of conventional optimization algorithms, such as their tendency to fall into local optima in high-dimensional spaces and difficulties in maintaining semantic consistency during fusion—this study enhances the original BKA through a series of systematic improvements, including adaptive step-size adjustment, elite memory with opposition-based perturbation, local Gaussian refinement, and diversity monitoring with reinitialization. These mechanisms dynamically balance global exploration and local exploitation, significantly improving stability and convergence for feature selection.

**Figure 1:**
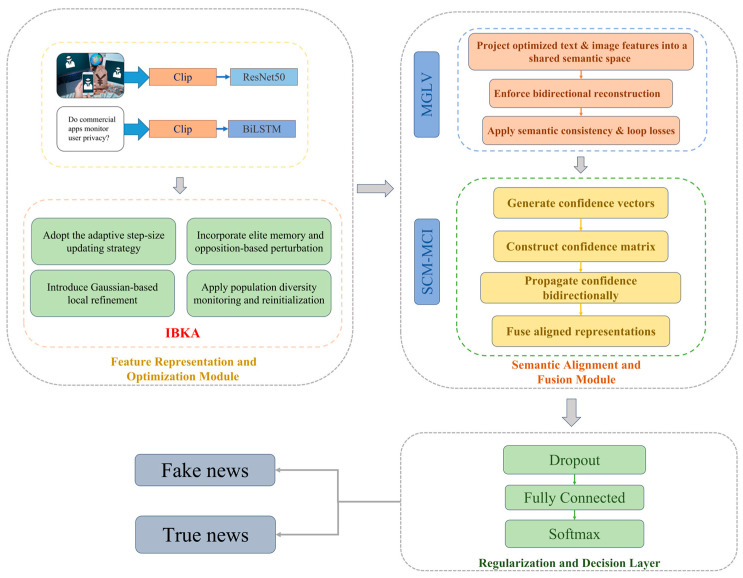
Framework of the IBKA-MSM multimodal fake news detection model.

Based on the improved algorithm, a Modality Generation and Loop Verification (MGLV) mechanism is developed to project different modalities into a shared semantic space through bidirectional semantic mapping and cyclic consistency constraints, thereby achieving global semantic alignment and complementary regularization. Furthermore, a Semantic Confidence Matrix with Modality-Cross Interaction mechanism (SCM-MCI) is designed to propagate and recalibrate semantic trust across modalities, enhancing the reliability and consistency of multimodal fusion. Overall, the IBKA-MSM framework employs a hierarchical pipeline of “optimization-driven feature selection—semantic alignment—confidence-based fusion,” ensuring interpretability while promoting stronger cross-modal reasoning capability and improved generalization performance in fake news detection tasks. The overall framework is illustrated in Figure 1. The collected original text has been translated into English for display.

### 3.1. Feature Extraction

#### 3.1.1. Text Feature Extraction

The textual modality primarily conveys news semantics and contextual information. Given its sequential characteristics, capturing contextual dependencies is crucial. Therefore, a BiLSTM-based sequential modeling approach is adopted to effectively learn bidirectional semantic relationships and extract text features enriched with contextual semantics.

Given an input text sequence $X_{t}=\left[x_{1},x_{2},\ldots ,x_{L}\right]$, it is first mapped into a word embedding matrix through an embedding layer.

(1) $$E_{t}=\mathrm{Embed}\left(X_{t}\right)\in R^{L\times D_{e}}$$

where L denotes the sentence length and $D_{e}$ is the embedding dimension.

The embedded sequence is then fed into a bidirectional long short-term memory network (BiLSTM) for contextual encoding.

(2) $$\vec{h_{t}}={\mathrm{LSTM}}_{f}\left(E_{t}\right)$$

(3) $$\overset{\leftarrow }{h_{t}}={\mathrm{LSTM}}_{b}\left(E_{t}\right)$$

The concatenation of forward and backward hidden states forms the bidirectional semantic representation.

(4) $$H_{t}=\left[\vec{h_{t}};\overset{\leftarrow }{h_{t}}\right]\in R^{L\times D_{h}}$$

where $D_{h}$ is the hidden dimension of each direction.

To obtain the global semantic representation of the entire text, average pooling is applied over all time steps.

(5) $$T_{f}=\mathrm{MeanPool}\left(H_{t}\right)\in R^{D_{t}}$$

The extracted feature vector $T_{f}$ comprehensively captures contextual dependencies and semantic consistency, thereby enhancing the capability of semantic discrimination in fake news detection.

#### 3.1.2. Image Feature Extraction

To fully capture the multi-level information within the visual modality, a dual-branch feature extraction strategy combining ResNet50 and CLIP is adopted. Structural and semantic features are extracted separately and then fused at the feature level to form a unified visual representation.

Given an input image $I\in R^{H\times W\times 3}$, a pre-trained ResNet50 is first used to extract convolutional structural features.

(6) $$F_{v}^{\left(r\right)}=\mathrm{ResNet}50\left(I\right)\in R^{D_{r}}$$

where $D_{r}$ denotes the dimension after global average pooling in the final layer of ResNet50, these features preserve spatial hierarchy and texture cues, enabling the intense discrimination of object boundaries and local patterns.

Next, the visual encoder of CLIP (Contrastive Language–Image Pre-training), based on the ViT-B/32 architecture, is employed to extract cross-modal semantic features.

(7) $$F_{v}^{\left(c\right)}={\mathrm{CLIP}}_{\mathrm{visual}}\left(I\right)\in R^{D_{c}}$$

$D_{c}$ is the visual semantic embedding dimension. Since CLIP is trained with cross-modal contrastive supervision, its visual features naturally align with language semantics and capture high-level conceptual cues.

Finally, the structural and semantic features are concatenated to form the complete visual representation.

(8) $$V_{f}=\left[F_{v}^{\left(r\right)};F_{v}^{\left(c\right)}\right]\in R^{D_{v}}$$

where $D_{v}=D_{r}+D_{c}$. The operator $\left[\;\cdot \;;\cdot \;\right]$ denotes feature-level concatenation.

This dual-branch strategy concurrently exploits low-level structural details and high-level semantic embeddings, thereby enhancing the integrity of visual representations and establishing a more discriminative foundation for subsequent feature selection and semantic alignment.

### 3.2. Black-Winged Kite Algorithm (BKA)

The Black-Winged Kite Algorithm (BKA) is a bio-inspired swarm intelligence optimization approach that emulates the cooperative predation patterns of black-winged kites in nature. By modeling behavioral processes such as surrounding, pursuing, and striking prey, BKA effectively maintains a balance between global search exploration and local solution refinement. The algorithm operates through three major phases: prey encircling, prey tracking, and prey attacking, which jointly drive adaptive optimization in complex search environments.

#### 3.2.1. Encircling the Prey

At the beginning of hunting, black kites circle the prey, adjusting their flight trajectories to determine the prey’s location and gradually narrowing the encirclement to make escape difficult. BKA mathematically models this behavior by treating each kite as a search agent and the prey position as the current optimal solution. During iterations, updates to movement are based on individual position and the prey’s location, formulated as.

(9) $$D=\left|C\cdot X^{*}\left(t\right)-X\left(t\right)\right|$$

(10) $$X\left(t+1\right)=X^{*}\left(t\right)-A\cdot D$$

where A  and C are coefficient vectors regulating the approaching speed, $X\left(t\right)$ denotes the individual position, and $X^{*}\left(t\right)$ represents the best-known prey position.

#### 3.2.2. Tracking the Prey

During the hunting process, black-winged kites continuously adjust their trajectories according to the prey’s estimated position, gradually narrowing the distance until capture is achieved. In the Black-Winged Kite Algorithm (BKA), each search agent updates its position based on the current best solution within the population, thereby improving global exploration and accelerating convergence. The update mechanism is formulated as.

(11) $$D_{i}=C_{i}\cdot \left(X^{*}\left(t\right)-X_{i}\left(t\right)\right)$$

(12) $$X_{i}\left(t+1\right)=X^{*}\left(t\right)-A_{i}\cdot D_{i}$$

where $X_{i}\left(t\right)$ represents the current position of the i-th kite, and $X_{i}^{*}\left(t\right)$ denotes the current best prey position found by the population.

The coefficients $A_{i}$ and $C_{i}$ control the movement amplitude and direction, respectively, and are defined as.

(13) $$A_{i}\left(t\right)=2a\left(t\right)r_{1,i}-a\left(t\right)$$

(14) $$\;C_{i}\left(t\right)=2r_{2,i}$$

(15) $$a\left(t\right)=a_{max}-\left(a_{max}-a_{min}\right)\frac{t}{T_{max}}$$

where $r_{1,i},r_{2,i}∼U\left(0,1\right)$, t is the current iteration, and $T_{max}$ is the maximum iteration number. The control parameter $a\left(t\right)$ decreases linearly from $a_{max}=2$ to $a_{min}=0$, ensuring that $\left|A_{i}\right|$ gradually decreases as iterations proceed. This mechanism encourages wide exploration in early stages and fine exploitation in later stages, maintaining population diversity while improving convergence stability.

#### 3.2.3. Attacking the Prey

Once the prey has been effectively surrounded and tracked, the black kites gradually tighten the encirclement and launch the final attack. In the BKA, this stage promotes a more exploitative search behavior by reducing the search radius and performing distance-controlled updates, allowing the population to converge precisely toward the global optimum.

Formally, for the i-th individual, the update rule in the attacking phase is defined as.

(16) $$X_{i}\left(t+1\right)=X^{*}\left(t\right)-A_{i}\cdot \left|C_{i}\cdot X^{*}\left(t\right)-X_{i}\left(t\right)\right|$$

In this formulation, $X_{i}\left(t\right)$ denotes the current position of the i-th search agent, while $X^{*}\left(t\right)$ represents the best-known prey location at iteration t. The coefficients $A_{i}$ and $C_{i}$ are adaptive control parameters that regulate the movement amplitude and direction of each agent. During the optimization process, the magnitude of $A_{i}$ decreases gradually. When $\left|A_{i}\right|<1$, the population performs fine-grained exploitation in the vicinity of the prey, enhancing local convergence precision. Conversely, when $\left|A_{i}\right|\geq 1$, agents maintain large exploratory movements to prevent premature entrapment in suboptimal regions.

### 3.3. Improved Black-Winged Kite Algorithm (IBKA)

The Black-Winged Kite Algorithm (BKA) exhibits rapid convergence and strong optimization capabilities, making it well-suited for solving feature selection tasks. Nevertheless, the original version reveals an imbalance between global exploration and local exploitation. During optimization, the search agents may quickly converge to a limited region of the search space, which restricts diversity and increases the risk of being trapped in local optima, thereby degrading overall performance. To overcome these limitations, this study introduces four enhancement strategies that extend the standard BKA and improve its robustness and solution quality.

#### 3.3.1. Introduce Dynamic Step-Length Regulation Mechanism

In the basic Black-Winged Kite Algorithm (BKA), the individual movement step size remains constant during the search process. This fixed-step mechanism may constrain the exploration scope at early stages, preventing the population from sufficiently covering the global search space. In the later phase, however, a considerable step size often leads to oscillation near the promising region, which undermines fine-grained local exploitation and slows convergence toward the optimal solution. To alleviate these issues, an adaptive step-size strategy is introduced. This mechanism dynamically adjusts the update amplitude according to the search progress, encouraging extensive exploration during initial iterations while emphasizing refined exploitation at later stages.

Specifically, let the current iteration number be t, the maximum iteration number be $T_{max}$, the position vector of the black kite individual be $X_{i}\left(t\right)$, and the optimal prey position be $X^{*}\left(t\right)$. The adaptive step size $\alpha \left(t\right)$ linearly decreases with the iterations, and its calculation formula is.

(17) $$\alpha \left(t\right)=\alpha_{max}-\frac{t}{T_{max}}\cdot \left(\alpha_{max}-\alpha_{min}\right)$$

where $\alpha_{max}$ and $\alpha_{min}$ are the maximum and minimum values of the step size, respectively, used to control the exploration amplitude in the early and later stages of the search. The step size decreases with the iterations, ensuring that the algorithm searches the global space with an extensive range in the initial stage and then shrinks the search in the later stages to achieve fine local exploitation. Based on the adaptive step size, the position update formula of individuals is as follows.

(18) $$X_{i}\left(t+1\right)=X_{i}\left(t\right)+\alpha \left(t\right)\cdot S_{i}\left(t\right)$$

where $S_{i}\left(t\right)$ is the direction vector of the individual update, which can be expressed as the weighted difference between the prey position and the current individual position.

(19) $$S_{i}\left(t\right)=r_{1}\cdot \left(X^{*}\left(t\right)-X_{i}\left(t\right)\right)+r_{2}\cdot \left(X_{rand}\left(t\right)-X_{i}\left(t\right)\right)$$

where $r_{1},r_{2}\in \left[0,1\right]$ are random disturbance coefficients used to increase randomness and diversity in the search. $X_{rand}\left(t\right)$ is the position of a randomly selected individual in the current population, used to introduce local exploration capability. In this way, each black kite individual not only approaches the optimal prey but can also conduct appropriate exploration within its global range, thereby preventing premature convergence of the population.

With the adoption of the adaptive movement scaling mechanism, the search agents can explore a more expansive solution space during the initial iterations and gradually shift to fine-grained exploitation as the optimization progresses. This dynamic adjustment enhances both the global exploration capability and the local convergence precision of the algorithm. The procedural implementation of this strategy is illustrated in Algorithm 1.


**Algorithm 1**: Adaptive Step-Size Update Strategy**Input:** Maximum/minimum step sizes $\alpha_{max}$, $\alpha_{min}$; maximum iteration $T_{max}$;        current iteration t; population X(t); best prey position $X^{*}\left(t\right)$.**Output:** Updated population matrix X(t+1).
1        Compute adaptive step size:

$\;\;\;\;\;\;\;\;\;\;\;\alpha \left(t\right)=\alpha_{max}-\frac{t}{T_{max}}\cdot \left(\alpha_{max}-\alpha_{min}\right)$

2        For each individual i in the population do3        Generate random coefficients r1, r2 ∼ U(0, 1).4        Select a random individual $X_{rand}\left(t\right)$ from the population5        Compute search direction:

$\;\;\;\;\;\;\;\;\;\;\;S_{i}\left(t\right)=r_{1}\cdot \left(X^{*}\left(t\right)-X_{i}\left(t\right)\right)+r_{2}\cdot \left(X_{rand}\left(t\right)-X_{i}\left(t\right)\right)$

6        Update position:

${\;\;\;\;\;\;\;\;\;\;\;X}_{i}\left(t+1\right)=X_{i}\left(t\right)+\alpha \left(t\right)\cdot S_{i}\left(t\right)$

7        (Optional) Apply boundary control to $X_{i}\left(t+1\right)$
8        End forEnd Procedure: Return updated population X(t+1).


#### 3.3.2. Integrate Elite Memory and Opposition-Based Perturbation Strategy

The elite memory mechanism can retain the positions of individuals that performed the best in history, guiding the current individuals toward excellent regions, thereby accelerating convergence and improving local exploitation accuracy. The opposition-based perturbation strategy generates opposite search vectors, allowing the population to maintain exploration diversity during the local convergence stage and effectively avoid falling into a local optimum. The combination of the two significantly enhances the global search ability and robustness of the algorithm, while also addressing the defect that the original BKA is prone to premature convergence in complex problems.

##### Elite Memory Mechanism

Let the historically best K individual positions be stored during the iteration process: $X_{elite}=\{{X_{elite}}^{1},{X_{elite}}^{2},\ldots ,{X_{elite}}^{k}\}$. When updating the current individual position, the guidance of elite memory is introduced. The specific update formula is:

(20) $$X_{i}^{elite}\left(t+1\right)=X_{i}\left(t\right)+\beta \cdot \left({X_{elite}}^{k}-X_{i}\left(t\right)\right)\cdot r$$

where ${X_{elite}}^{k}$ is a randomly selected elite individual position, $\beta \in \left(0.1\right)$ is the elite guidance coefficient, and $r\in \left[0,1\right]$ is the random disturbance coefficient used to increase search diversity. This formula enables individuals to approach historically excellent solutions while retaining their original update direction, improving convergence stability.

##### Opposition-Based Perturbation Strategy

To avoid premature convergence and reduce the likelihood of stagnation in local optima, opposition-based perturbation is incorporated into the optimization process. This strategy generates candidate solutions on the opposite side of the current position, thereby enhancing search diversity and improving the probability of reaching a better region of the solution space. Let the current individual position be $X_{i}\left(t\right)$, and the lower and upper bounds of the search space be $\left[X_{min},X_{max}\right]$, then the opposite position can be expressed as.

(21) $$X_{i}^{op}\left(t\right)=X_{min}+X_{max}-X_{i}\left(t\right)$$

Combining random disturbance $\epsilon ∼U\left(0,1\right)$, the final update formula is:

(22) $$X_{i}\left(t+1\right)=X_{i}^{elite}\left(t+1\right)+\gamma \cdot \epsilon \cdot \left(X_{i}^{op}\left(t\right)-X_{i}^{elite}\left(t+1\right)\right)$$

where $\gamma \in \left(0,1\right)$ is the perturbation coefficient. By incorporating an opposite search direction into the elite guidance, individuals have a higher chance of escaping the local optimum and enhancing their global search capabilities.

#### 3.3.3. Apply Local Gaussian Refinement Strategy

During the optimization process of the Black-Winged Kite Algorithm, even with the introduction of elite memory and opposition-based perturbation, individuals may still stagnate near the elite mask boundary, resulting in insufficient local search precision. To further enhance the local exploitation ability, a local Gaussian refinement strategy is introduced. The strategy performs perturbation search near the elite mask boundary of elite individuals to enhance nuanced exploration of the optimal solution.

Let the current elite individual position be ${X_{elite}}^{k}$. In its neighborhood, a Gaussian perturbation is performed on some feature dimensions.

(23) $$X_{i}^{gauss}\left(t+1\right)=X_{i}\left(t+1\right)+\sigma \cdot N\left(0,1\right)\cdot M_{elite}$$

where σ>0 is the standard deviation controlling the perturbation amplitude, and $N\left(0,1\right)$ denotes a standard normally distributed random variable.

The elite mask vector $M_{\mathrm{elite}}$ identifies the feature dimensions eligible for perturbation and is defined as follows:

(24) $${{(M}_{elite})}_{d}=\left\{\begin{array}{c} 1,if\left|x_{d}^{elite}-\mu_{d}\right|\leq \tau \sigma_{d}\\ 0,\mathrm{otherelse} \end{array}\right.$$

Here, $x^{elite}$ is the best individual or the mean of top-k elites, $\mu_{d}$ and $\sigma_{d}$ are the mean and standard deviation of the d-th dimension across the population, and $\tau \in \left[1.0,1.5\right]$ is a threshold controlling the selection width of elite dimensions.

This mask limits perturbation to near-optimal regions, enhancing fine-grained search accuracy while maintaining stability and diversity.

#### 3.3.4. Implement Diversity Monitoring and Reinitialization Strategy

In the original Black-Winged Kite Algorithm, individual updates rely mainly on historical positions and random perturbations, without explicit monitoring of population diversity. As iterations proceed, individuals may cluster prematurely in local regions, reducing diversity, weakening global exploration, and increasing the likelihood of being trapped in local optima.

To address this issue, a diversity monitoring and reinitialization strategy is proposed. The diversity of the population is dynamically evaluated, and when stagnation is detected, low-activity individuals are reinitialized locally or globally to restore diversity and enhance robustness.

Let the population contain N individuals, each represented by $X_{i}\left(t\right)$. The population diversity index is defined as:

(25) $$\Delta \left(t\right)=\frac{1}{N}\sum_{i=1}^{N}∥X_{i}\left(t\right)-\bar{X}\left(t\right){∥}_{2}$$

(26) $$\bar{X}\left(t\right)=\frac{1}{N}\sum_{i=1}^{N}X_{i}\left(t\right)$$

The minimum diversity threshold is expressed as:

(27) $$D_{min}\left(t\right)=\kappa \cdot {median}_{i<j}\left(∥X_{i}\left(t\right)-X_{j}\left(t\right){∥}_{2}\right)$$

where $\kappa \in \left[0.25,0.40\right]$ is a scaling factor (typically κ=0.3). When $\Delta \left(t\right)<D_{min}\left(t\right)$, the population diversity is considered too low, and reinitialization is triggered to prevent premature convergence.

The reinitialization rule is given by:

(28) $$X_{i}\left(t+1\right)=\left\{\begin{array}{c} L+\left(U-L\right)\cdot Tent\left(\mu \right),with\;probability\;0.5\\ L+U-X_{i}\left(t\right),with\;probality\;0.5 \end{array}\right.$$

where L and U denote the lower and upper bounds of the search space, and $\mathrm{Tent}\left(u\right)$ is the chaotic Tent map defined as:

(29) $$Tent\left(\mu \right)=\left\{\begin{array}{c} 2\mu ,\mu <0.5,\\ 2(1-\mu ),\mu \geq 0.5, \end{array}\mu \sim U\left(0,1\right)\right.$$

This hybrid chaotic–oppositional initialization combines random reallocation and mirrored opposition to reintroduce diversity effectively.

Through this adaptive mechanism, the population can dynamically adjust its distribution, maintain exploratory capability, and avoid premature convergence, thereby improving both global search efficiency and robustness.

The pseudocode of this strategy is shown in Algorithm 2.


**Algorithm 2**: Diversity Monitoring and Reinitialization Strategy in IBKAInput: Population size N; individual positions $X_{i}\left(t\right)$; bounds L, U; scaling factor κ;           Tent map.Output: Updated population X(t+1).
1        Compute the population mean:

$\;\;\;\;\;\;\;\;\;\;\;\bar{X}\left(t\right)=\frac{1}{N}\sum_{i=1}^{N}X_{i}\left(t\right)$

2        Compute the diversity index:

$\;\;\;\;\;\;\;\;\;\;\;\;\Delta \left(t\right)=\frac{1}{N}\sum_{i=1}^{N}∥X_{i}\left(t\right)-\bar{X}\left(t\right){∥}_{2}$

3        Compute minimum diversity threshold:

$\;\;\;\;\;\;\;\;\;\;\;D_{min}\left(t\right)=\kappa \cdot {median}_{i<j}\left(∥X_{i}\left(t\right)-X_{j}\left(t\right){∥}_{2}\right)$

4        If $\Delta \left(t\right)<D_{min}\left(t\right),$ diversity is too low → Trigger reinitialization.5        For each individual i=1,2,…,N do6        Generate $\mu \sim U\left(0,1\right)$.7        Compute $Tent\left(\mu \right)$:

$\;\;\;\;\;\;\;\;\;\;\;\;Tent\left(\mu \right)=\left\{\begin{array}{c} 2\mu ,\mu <0.5\\ 2(1-\mu ),\mu \geq 0.5 \end{array}\right.$

8        with probability 0.5:

${\;\;\;\;\;\;\;\;\;\;\;\;X}_{i}\left(t+1\right)=L+\left(U-L\right)\cdot Tent\left(\mu \right)$

9        with probality 0.5:

${\;\;\;\;\;\;\;\;\;\;\;\;X}_{i}\left(t+1\right)=L+U-X_{i}\left(t\right)$

10        End for11        Else12        $X_{i}\left(t+1\right)=X_{i}(t)$, for all i=1,2,…,N
13        End ifEnd Procedure: Return updated population X(t+1).


To further improve stability, the proposed diversity monitoring strategy is also linked with an adaptive convergence mechanism. Instead of relying solely on the maximum iteration number as a stopping criterion, the algorithm continuously monitors the change rate of the global best fitness value. When the improvement in fitness remains below a small threshold  ϵ for a predefined number of successive iterations $T_{\mathrm{stagn}}$, the population is considered stagnant. In such cases, the diversity reinitialization and local Gaussian refinement processes are automatically activated to refresh part of the population and continue exploration. This adaptive mechanism effectively prevents premature termination and ensures that the algorithm continues searching toward the global optimum even when approaching the iteration limit.

### 3.4. Unified Modality Space and Semantic Alignment

Although the multimodal features optimized by IBKA have achieved remarkable improvements in discriminability and compactness, discrepancies in semantic structure and feature distributions across different modalities still exist. Visual features typically exhibit spatial semantic relationships, whereas textual features emphasize sequential contextual dependencies. Such disparities in semantic spaces make direct fusion prone to information bias and modality mismatch, thereby weakening the expression of cross-modal semantic associations. To achieve unified modality representation and distribution convergence, a feature alignment module is constructed on top of the IBKA-optimized features. The module introduces a Modality Generation and Loop Verification mechanism (MGLV) to enable semantic-level cross-modal mapping and alignment.

#### 3.4.1. Modality Generation and Loop Verification Mechanism (MGLV)

In multimodal semantic modeling, textual and visual features are usually distributed in heterogeneous representation spaces. The former emphasizes semantic sequence and contextual dependency, while the latter focuses on spatial structure and local visual patterns. This semantic-level discrepancy can easily lead to a semantic shift and distribution mismatch during cross-modal feature fusion. To achieve global cross-modal semantic alignment and complementary constraints, this study designs the Modality Generation and Loop Verification mechanism (MGLV) on top of the IBKA-optimized features. Through semantic subspace projection and bidirectional consistency reconstruction, this mechanism provides comparable and reversible representational forms in a shared semantic space, thereby enabling high-level alignment across modalities.

Let the IBKA-optimized textual and visual features be represented as:

(30) $$X^{\left(T\right)}={\left[x_{1}^{\left(T\right)},x_{2}^{\left(T\right)},\ldots ,x_{N}^{\left(T\right)}\right]}^{⊤}\in R^{N\times D_{T}}$$

(31) $$X^{\left(I\right)}={\left[x_{1}^{\left(I\right)},x_{2}^{\left(I\right)},\ldots ,x_{N}^{\left(I\right)}\right]}^{⊤}\in R^{N\times D_{I}}$$

where N is the number of samples, and $D_{T}$ and $D_{I}$ denote the original feature dimensions of textual and visual modalities, respectively. To eliminate dimensional discrepancies, MGLV applies randomly initialized linear projection matrices $W_{T}\in R^{D_{T}\times D_{P}},W_{I}\in R^{D_{I}\times D_{P}}$ to map both modalities into a shared semantic space $R^{D_{P}}$:

(32) $$h_{n}^{\left(T\right)}=x_{n}^{\left(T\right)}W_{T}$$

(33) $$h_{n}^{\left(I\right)}=x_{n}^{\left(I\right)}W_{I}$$

To avoid scale-induced semantic shifting, the projected vectors are normalized:

(34) $${\hat{h}}_{n}^{\left(T\right)}=\frac{h_{n}^{\left(T\right)}}{∥h_{n}^{\left(T\right)}{∥}_{2}+\varepsilon }$$

(35) $${\hat{h}}_{n}^{\left(I\right)}=\frac{h_{n}^{\left(I\right)}}{∥h_{n}^{\left(I\right)}{∥}_{2}+\varepsilon }$$

where $∥\cdot {∥}_{2}$ denotes the Euclidean norm and ε>0 ensures numerical stability. The cosine similarity between modalities in the projection space is then computed:

(36) $$s_{n}={\hat{h}}_{n}^{\left(T\right)⊤}{\hat{h}}_{n}^{\left(I\right)},\;s_{n}\in \left[0,1\right]$$

(37) $$\bar{s}=\frac{1}{N}\sum_{n=1}^{N}s_{n}$$

where $\bar{s}$ measures the overall semantic consistency of the shared feature space. To ensure reversible alignment, a loop verification mechanism is introduced. Text representations are reconstructed via the visual pathway.

(38) $$r_{n}^{\left(T\right)}=\left({{\hat{h}}_{n}^{\left(I\right)}W}_{I}^{⊤}\right)W_{T}$$

Similarly, visual representations are reconstructed via the textual pathway.

(39) $$r_{n}^{\left(I\right)}=\left({{\hat{h}}_{n}^{\left(T\right)}W}_{T}^{⊤}\right)W_{I}$$

The corresponding loop consistency loss is defined as:

(40) $$L_{\mathrm{loop}}=\frac{1}{N}\sum_{n=1}^{N}\left(∥r_{n}^{\left(T\right)}-{\hat{h}}_{n}^{\left(T\right)}{∥}_{2}^{2}+∥r_{n}^{\left(I\right)}-{\hat{h}}_{n}^{\left(I\right)}{∥}_{2}^{2}\right)$$

To prevent over-parameterization and enhance generalization, a Frobenius regularization is applied.

(41) $$R=∥W_{T}{∥}_{F}^{2}+∥W_{I}{∥}_{F}^{2}$$

where $∥\cdot {∥}_{F}$ denotes the Frobenius norm of a matrix, which is used to constrain the overall magnitude of the weights in the projection matrices. Thus, the MGLV objective function is formulated as:

(42) $$L_{\mathrm{MGLV}}=\left(1-\bar{s}\right)+\lambda_{\mathrm{loop}}L_{\mathrm{loop}}+\lambda_{\mathrm{reg}}R$$

where $\lambda_{\mathrm{loop}}$ and $\lambda_{\mathrm{reg}}$ are hyperparameters used to balance loop consistency and model complexity.

Through this mechanism, MGLV accomplishes reversible and stable cross-modal semantic alignment without requiring additional supervision, providing a unified and robust modality representation backbone for subsequent modeling.

#### 3.4.2. Semantic Confidence Matrix and Modality-Cross Interaction (SCM-MCI)

After completing the modality generation–loop verification mechanism, the textual and visual modalities have achieved global semantic alignment in the shared semantic subspace. Due to differences in information granularity, abstraction level, and semantic saliency between the two modalities, subtle uncertainties and semantic deviations may still exist. To further enhance the information complementarity and reliability discrimination capability across modalities, this study introduces a Semantic Confidence Matrix and Modality-Cross Interaction mechanism (SCM-MCI) based on the MGLV-aligned features.

Specifically, let the normalized textual and visual features after MGLV be denoted as $r_{n}^{\left(T\right)}\in R^{D_{P}},r_{n}^{\left(I\right)}\in R^{D_{P}}$, respectively, where $D_{P}$ is the dimensionality of the shared semantic space. To represent the reliability of each modality under the current semantic context, modality confidence vectors are defined as:

(43) $$c_{n}^{\left(T\right)}=\sigma \left(W_{c}^{\left(T\right)}r_{n}^{\left(T\right)}+b_{c}^{\left(T\right)}\right)$$

(44) $$c_{n}^{\left(I\right)}=\sigma \left(W_{c}^{\left(I\right)}r_{n}^{\left(I\right)}+b_{c}^{\left(I\right)}\right)$$

where $\sigma \left(\cdot \right)$ is the Sigmoid activation function used to constrain confidence values within the range [0, 1], $W_{c}^{\left(T\right)},W_{c}^{\left(I\right)}\in R^{D_{P}\times D_{P}}$ are learnable confidence mapping matrices, and $b_{c}^{\left(T\right)},b_{c}^{\left(I\right)}$ are the corresponding bias vectors.

This structure automatically assigns weights based on the local semantic characteristics of each sample, thereby identifying modality dimensions that contribute more strongly to the semantic content. Here, $c_{n}^{\left(T\right)}$ and $c_{n}^{\left(I\right)}$ can be regarded as “semantic confidence responses,” reflecting the semantic certainty possessed by each modality in the discrimination task. After obtaining the confidence vectors, a sample-level semantic confidence matrix is constructed to further model semantic interaction between the two modalities.

(45) $$M_{n}=c_{n}^{\left(T\right)}{\left(c_{n}^{\left(I\right)}\right)}^{⊤}$$

where $M_{n}\in R^{D_{P}\times D_{P}}$ characterizes the degree of confidence coupling across semantic dimensions of text and image in the shared space. A larger value of $M_{n}\left(i,j\right)$ indicates a stronger semantic contribution consistency between the i-th textual dimension and the j-th visual dimension. In comparison, a smaller value indicates weaker involvement in cross-modal interaction. Based on $M_{n}$, the SCM-MCI module performs dynamic interaction updates on the aligned features to propagate high-confidence semantic information between modalities.

(46) $${\tilde{r}}_{n}^{\left(T\right)}=r_{n}^{\left(T\right)}+\alpha M_{n}r_{n}^{\left(I\right)}$$

(47) $${\tilde{r}}_{n}^{\left(I\right)}=r_{n}^{\left(I\right)}+\alpha M_{n}^{⊤}r_{n}^{\left(T\right)}$$

where $\alpha \in \left(0,1\right]$ is the interaction balance coefficient used to control semantic propagation strength. This bidirectional updating process can be viewed as a semantic confidence propagation mechanism. The mechanism enhances high-confidence regions across different modalities while suppressing low-confidence regions, thereby achieving confidence-based cross-modal semantic recalibration. After the bidirectional update, the interacted modality features are fused as follows.

(48) $$r_{n}^{\left(F\right)}=\gamma_{1}{\tilde{r}}_{n}^{\left(T\right)}+\gamma_{2}{\tilde{r}}_{n}^{\left(I\right)}+\gamma_{3}\left({\tilde{r}}_{n}^{\left(T\right)}⊙{\tilde{r}}_{n}^{\left(I\right)}\right)$$

where ⊙ denotes the Hadamard element-wise product, and $\gamma_{1},\gamma_{2},\gamma_{3}$ are learnable fusion coefficients used to balance contributions from different fusion pathways.

This structure combines linear composition with non-linear interactions, enabling the fused features to preserve modality-specific semantics while capturing deeper semantic synergy. To ensure numerical stability and generalization of the confidence modeling process, a confidence regularization term is introduced.

(49) $$L_{\mathrm{conf}}=\frac{1}{N}\sum_{n=1}^{N}(∥c_{n}^{\left(T\right)}-c_{n}^{\left(I\right)}{∥}_{2}^{2}+∥M_{n}{∥}_{F}^{2})$$

where $∥\cdot {∥}_{2}$ denotes the vector L2-norm that measures similarity between modality confidence distributions, and $∥\cdot {∥}_{F}$ denotes the Frobenius norm used to constrain the magnitude of $M_{n}$ to prevent over-coupling.

By integrating the above mechanisms, the overall optimization objective of the SCM-MCI module is expressed as.

(50) $$L_{\mathrm{SCM}\text{-}\mathrm{MCI}}=L_{\mathrm{MGLV}}+\lambda_{\mathrm{conf}}L_{\mathrm{conf}}$$

where $\lambda_{\mathrm{conf}}$ is a balancing coefficient used to adjust the trade-off between semantic alignment and confidence regularization.

Through this joint optimization, SCM-MCI achieves unified semantic confidence modeling, information interaction, and structural consistency constraints in the shared semantic space. Additionally, SCM-MCI provides the model with enhanced robustness and interpretability when handling multimodal uncertainty. This mechanism not only strengthens semantic complementarity across modalities but also promotes the transformation from “alignment” to “confidence-aware interaction” during fusion, offering more stable semantic support for subsequent feature fusion and classification.

## 4. Experimental Analysis

### 4.1. Data Collection

The data used in this study are obtained from three authoritative information sources: Sina News, Science China, and the China Internet Joint Rumor-Refutation Platform, resulting in a total of 7538 textual samples and 6262 image samples. Among them, Sina News provides 2449 real news items and 1260 associated images, covering multiple domains, including society, finance, and technology. Science China contributes 4186 fake news items and 4100 images, primarily focusing on the dissemination of scientific knowledge and the refutation of pseudoscientific content. The China Internet Joint Rumor-Refutation Platform contributes 903 fake news items and 902 images, primarily targeting rumor identification and multi-source information verification. The sample distribution across the three platforms is summarized in Table 1.

To ensure consistency and independence of samples during the training, validation, and testing stages, a stratified sampling strategy is adopted for three-stage data partitioning. First, the entire dataset is randomly divided into a training set and a test set at a ratio of 7:3, ensuring that test samples remain completely isolated during training and are only used for final performance evaluation. Subsequently, 20% of the training set is further divided into a validation set to enable hyperparameter tuning and early stopping during model optimization, thereby preventing overfitting. Through this two-step process, the final proportions of the training, validation, and test sets are 56%, 14%, and 30%, respectively.

### 4.2. Evaluation Metrics

To comprehensively evaluate the model’s performance in multimodal fake news detection, four commonly used classification metrics are employed in the experiments: Accuracy, Precision, Recall, and F1-score. Accuracy measures the overall correctness of classification. Precision reflects the reliability of predicting fake news. Recall indicates the model’s capability of identifying fake news samples, and F1-score considers the balance between Precision and Recall, serving as an overall measure under class-imbalanced conditions [33,34,35]. The formulas for the four metrics are defined as follows:

(51) $$\mathrm{Accuracy}=\frac{TP+TN}{TP+TN+FP+FN}$$

(52) $$\mathrm{Precision}=\frac{TP}{TP+FP}$$

(53) $$\mathrm{Recall}=\frac{TP}{TP+FN}$$

(54) $$F1\text{-}\mathrm{score}=\frac{2\times \mathrm{Precision}\times \mathrm{Recall}}{\mathrm{Precision}+\mathrm{Recall}}$$

Among them, TP (True Positive) denotes the number of fake news samples correctly identified. TN (True Negative) denotes the number of real news samples correctly identified. FP (False Positive) represents the number of real news samples mistakenly classified as fake, and FN (False Negative) represents the number of fake news samples incorrectly classified as real. In the specific experiments, Accuracy reflects the overall discrimination capability of the model. Precision measures its reliability in identifying fake news. Recall reflects the model’s sensitivity in capturing fake news samples. and the F1-score serves as a comprehensive metric to balance Accuracy and recall performance. To ensure stability and comparability of the results, all metrics are calculated based on predictions on the test set, and a unified evaluation is performed across all comparison models.

### 4.3. Experimental Settings

Regarding the hyperparameter configuration, all parameters are tuned using the validation set. During training, adaptive optimization and regularization strategies are employed to strike a balance between convergence speed and generalization performance. Core parameters, including embedding dimensions, transformer architecture size, learning rate, and regularization coefficients, are selected based on a comprehensive evaluation of multiple experimental trials. The final configurations are summarized in Table 2. To prevent overfitting, early stopping and dropout are applied during training, and the learning rate is dynamically adjusted with a cosine annealing strategy, thereby improving the overall model performance while maintaining training stability.

### 4.4. Multimodal Comparison Experiments

To comprehensively evaluate the discrimination capability and generalization performance of the proposed IBKA-MSM framework in multimodal fake news detection, several representative state-of-the-art multimodal detection models are introduced as comparison baselines, including EANN, SAFE, MVAE, MCAN, MMFakeBuster, and MDFEND. These models cover typical paradigms of modality alignment, feature fusion, and cross-modal interaction. All models are trained under the same feature inputs, data partitioning, and training strategy to ensure fairness and comparability of the results. The performance comparison of all models is reported in Table 3. The bold indicates the best performance.

EANN: achieves cross-event fake news detection through event-invariant adversarial feature learning, demonstrating good transfer generalization capability; however, its ability to capture complex text–image associations is limited.

SAFE [36]: explicitly models text–image similarity differences using a dual-branch attention mechanism, which effectively identifies semantic inconsistency, but exhibits degraded performance when handling highly similar forged samples.

MVAE [37]: conducts joint text–image modeling based on shared latent variables and is robust to modality missing or noisy conditions; however, its capability of modeling deep interaction features remains insufficient.

MCAN [38]: enhances inter-modal dependency with a multi-layer co-attention structure but suffers from high computational complexity and limited generalization under low-correlation samples.

MMFakeBuster [39]: improves multimodal cooperative representation through lightweight residual fusion and demonstrates high training efficiency, yet the shallow fusion structure restricts its ability to handle cross-modal semantic transfer.

MDFEND [40]: captures emotional cues via dual-path emotional modeling and contrastive learning, showing strong performance on emotion-driven fake content.

As shown in Table 3, IBKA-MSM exhibits significant advantages across all performance metrics. In the four core indicators, the model’s overall performance surpasses that of existing baseline methods, exhibiting superior stability and balance. Compared to the best-performing baseline MCAN, the Accuracy of IBKA-MSM improves by approximately 3.0%, while Precision and Recall increase by 2.3% and 2.1%, respectively. The F1-score reaches 94.14%, achieving an ideal balance between precision and Recall. These results not only demonstrate improvements in overall classification capability but also imply stronger expressiveness and adaptability in complex semantic alignment and cross-modal interaction modeling.

Further analysis reveals that IBKA-MSM effectively avoids overfitting to the majority class while maintaining high accuracy. The high Recall indicates that the model’s ability to identify minority-class samples (fake news) is significantly enhanced. In comparison, earlier models such as SAFE and EANN are capable of capturing shallow cross-modal semantic relationships but remain insufficient in deep semantic consistency modeling and dynamic modality complementation. Although MDFEND and MVAE introduce improvements in modality alignment mechanisms, their global optimization and noise suppression abilities in the feature space are still limited. MMFakeBuster and MCAN possess relatively strong fusion structures, yet may still encounter feature redundancy or single-modality reliance issues under complex multimodal semantic scenarios. In contrast, IBKA-MSM significantly enhances the discriminability and complementarity of modality features through the improved Black-Winged Kite Optimization algorithm and multi-strategy collaborative modeling, thereby maintaining robust performance across different scenarios.

Moreover, the adaptive step size and elite memory mechanisms employed in IBKA-MSM achieve a dynamic balance between global exploration and local exploitation during feature search, effectively overcoming the limitation of conventional swarm intelligence algorithms that easily fall into local optima. This feature optimization process not only improves the effectiveness of modality features but also enhances model interpretability and robustness through refined feature selection and weight adjustment. Meanwhile, the multi-strategy collaborative modeling framework achieves deep semantic alignment in the fusion stage, enabling text and image modalities to form complementary representational structures in the high-dimensional space. This enhancement further improves the model’s ability to capture implicit cross-modal consistency.

Overall, IBKA-MSM demonstrates outstanding comprehensive performance in multimodal fake news detection. The model not only achieves superior results across quantitative metrics but also exhibits unique innovation and methodological value in key aspects such as feature optimization, semantic fusion, and cross-modal collaborative modeling.

### 4.5. Comparative Experiments of Meta-Heuristic Algorithms

To evaluate the effectiveness of different swarm intelligence algorithms in multimodal feature selection and fusion, four representative nature-inspired optimization methods are considered in this study, including Whale Optimization Algorithm (WOA), Particle Swarm Optimization (PSO) [41], Black-Winged Kite Algorithm (BKA), and the Improved Black-Winged Kite Algorithm (IBKA). All these algorithms are based on principles of population intelligence and perform global exploration and local exploitation within the feature subspace to search for the optimal feature subset, thereby enhancing the discriminative capability of multimodal fake news detection. The detection performance of these four algorithms integrated with the MSM framework is presented in Table 4. The bold indicates the best performance.

The results show that the WOA-MSM model achieves an accuracy of 66.92% and an F1-score of 46.18%, primarily due to its excessively rapid early convergence and insufficient global exploration during the feature selection stage. PSO-MSM enhances search stability through the velocity update mechanism, resulting in a significant performance improvement, with an accuracy of 96.48% and an F1-score of 91.38%. However, it still easily encounters local oscillations when dealing with high-dimensional feature spaces. BKA-MSM achieves a good balance between precision and recall, with a precision of 91.58%, a recall of 93.55%, and an F1-score of 92.55%. Its hybrid perturbation mechanism helps maintain search activity during later iterations. Although IBKA-MSM shows a slightly lower accuracy, it obtains the highest F1-score of 94.14% due to the high stability brought by adaptive step-size and chaotic initialization, indicating that the improvement strategies effectively enhance model generalization and robustness.

The observed decrease in accuracy after the proposed improvements can be attributed to the introduction of stronger regularization and diversity mechanisms in the model. These enhancements aim to improve the model’s robustness and generalization ability rather than maximizing accuracy on a single dataset. As a result, the model becomes less sensitive to specific data patterns or noise, which may lead to a slight decline in accuracy but ensures more stable and reliable performance across varying data distributions.

Overall, IBKA-MSM demonstrates the most substantial advantages in stability and balance, showing strong robustness and application potential in multimodal fake news detection tasks, and providing an important reference for designing future multimodal optimization frameworks.

### 4.6. Convergence Performance Analysis of the IBKA

To systematically verify the global optimization capability and convergence characteristics of the Improved Black-Winged Kite Algorithm (IBKA), comparative experiments are conducted on six classical continuous optimization benchmark functions widely adopted in the CEC benchmark suite, including Griewank [42], Ackley [43], Sum Squares [44], Rosenbrock [45], Levy, and Sphere functions [46]. These functions are standard test problems in the field of meta-heuristic optimization and are used to evaluate different dimensions of algorithmic performance. The Sphere and Sum Squares functions belong to unimodal benchmark functions that assess convergence accuracy and exploitation capability. The Griewank, Ackley, and Levy functions are multimodal functions that test global exploration ability and robustness against local optima. The Rosenbrock function represents a narrow-valley optimization problem designed to measure convergence stability in nonlinear, high-dimensional landscapes.

**Figure 2:**
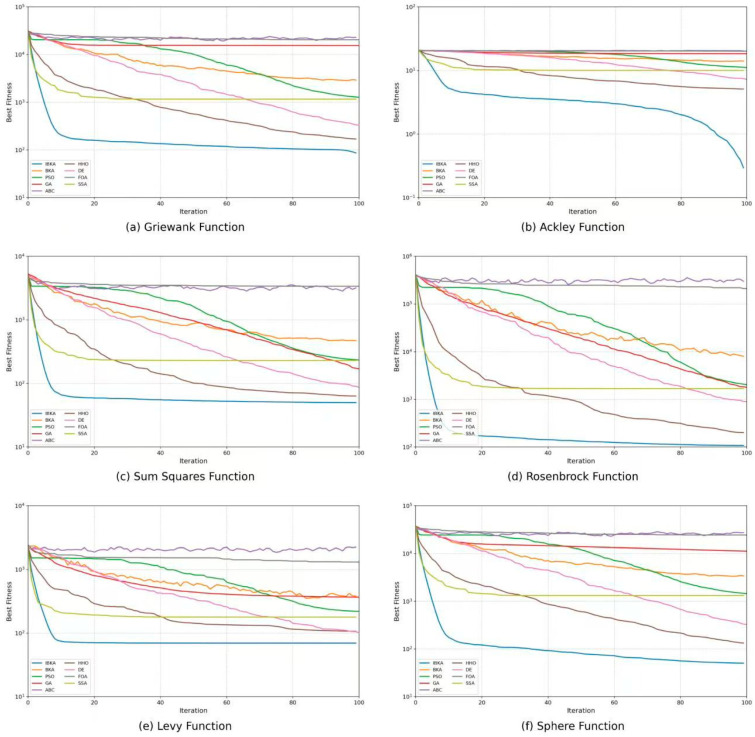
Convergence curve comparison between IBKA and eight mainstream meta-heuristic optimization algorithms.

The comparison algorithms include the Black-Winged Kite Algorithm (BKA), Particle Swarm Optimization (PSO), Genetic Algorithm (GA) [47], Artificial Bee Colony (ABC) [48], Harris Hawks Optimization (HHO) [49], Differential Evolution (DE) [50], Fruit Fly Optimization Algorithm (FOA) [51], and Sparrow Search Algorithm (SSA). All algorithms are executed under identical experimental conditions, including the same population size, dimensionality, and maximum number of iterations. Each experiment is repeated 30 times, and the averaged fitness results are recorded to ensure statistical reliability and stability. The convergence results are illustrated in Figure 2.

As shown in Figure 2, IBKA exhibits a significantly superior convergence trend compared with other algorithms on all six benchmark functions. For the Griewank and Ackley functions, IBKA rapidly decreases the fitness value during the early iterations and reaches the stable convergence stage much earlier. In contrast, algorithms such as BKA, PSO, and GA maintain relatively high fitness levels in later stages. This result demonstrates that IBKA possesses stronger global exploration capability and a higher ability to escape local optima in multimodal environments. For the Sum Squares and Rosenbrock functions, IBKA not only converges faster but also achieves the lowest final objective value with smoother and more stable curves, reflecting its advantages in convergence accuracy and reliability within nonlinear high-dimensional spaces. Regarding the Levy function, IBKA shows minor fluctuations at the beginning but maintains a clear downward trend, and its final convergence value is markedly better than those of DE, FOA, and SSA, highlighting its robustness in rugged optimization landscapes. In the Sphere function, IBKA nearly reaches the optimal region within the first twenty iterations, demonstrating its high convergence efficiency and substantial precision in unimodal scenarios.

Overall, IBKA achieves the fastest convergence speed and the lowest final fitness value across all benchmark functions, outperforming traditional algorithms in terms of comprehensive performance. Its superiority mainly stems from the introduction of the adaptive step-size adjustment mechanism and elite memory strategy. The former dynamically balances global exploration and local exploitation based on the iteration process, while the latter preserves high-quality individual information to enhance solution diversity and global convergence accuracy. Therefore, IBKA demonstrates significant advantages in convergence speed, optimization stability, and global search capability, providing a solid algorithmic foundation for subsequent feature selection and multimodal optimization tasks.

### 4.7. Model Interpretability and Feature Visualization Analysis

**Figure 3:**
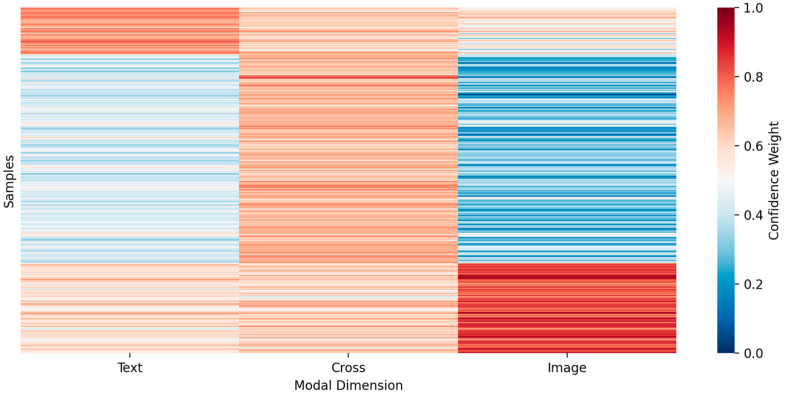
Semantic Confidence Distribution Learned by the SCM-MCI Module.

**Figure 4:**
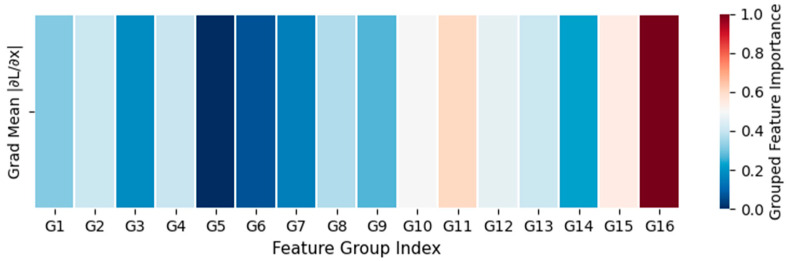
Grouped feature importance distribution of the Transformer classifier.

To further verify the internal operating mechanism of the SCM-MCI framework in multimodal fusion and decision-making, this section conducts interpretability visualization from two perspectives: semantic confidence distribution and feature importance. Figure 3 illustrates the confidence allocation across three semantic spaces—text, cross-modal, and image—revealing the model’s dynamic fusion strategy under varying modality contributions. Figure 4 presents the grouped feature importance of the Transformer classifier, which helps identify the discriminative semantic regions emphasized during classification.

As shown in Figure 3, the confidence distribution across the three modality spaces exhibits a smooth and continuous transition, indicating that the semantic consistency constraint effectively balances modality contributions and prevents excessive reliance on a single source of information. Furthermore, the relatively lower confidence assigned to the cross-modal dimension suggests a cautious alignment mechanism when handling heterogeneous semantic spaces, thus improving the stability and robustness of the decision-making process.

Figure 4 illustrates the distribution of grouped feature importance based on gradient sensitivity. It can be observed that most feature groups (G1–G14) contribute moderately and consistently to the prediction, demonstrating that the model comprehensively leverages global fused representations. In contrast, the markedly higher importance of G16 implies that the classifier is capable of focusing on highly discriminative fused semantic regions, which serve as critical cues for the final judgment.

Taken together, the two visual analyses confirm that the SCM-MCI framework not only maintains strong robustness and balance in multimodal fusion but also exhibits reliable semantic selectivity and interpretability. These results demonstrate the effectiveness of the proposed approach for cross-modal alignment and high-level semantic discrimination.

## 5. Conclusions and Future Work

### 5.1. Conclusions

This study focuses on the problems of cross-modal semantic inconsistency, insufficient feature correlation, and weak robustness of modality fusion in multimodal fake news detection tasks. It proposes a detection framework based on improved Black-Winged Kite optimization and multimodal semantic modeling, called IBKA-MSM. This framework constructs a systematic detection process from three levels: modality feature generation, semantic alignment, and deep fusion, achieving an organic integration of feature optimization, semantic modeling, and fusion decision-making.

In the feature generation stage, the proposed Improved Black-Winged Kite Algorithm (IBKA) is used for cross-modal feature generation and optimization. The algorithm incorporates an adaptive step-size update strategy, elite memory, opposition-based perturbation mechanisms, Gaussian-based local refinement, and a population diversity monitoring and re-initialization strategy into the original search mechanism, thereby achieving a dynamic balance between global exploration and local exploitation. This design effectively improves the discriminability and stability of features in the multimodal feature generation stage, providing high-quality modality inputs for subsequent semantic modeling.

In the semantic alignment stage, the designed Modality Generation–Loop Verification (MGLV) mechanism focuses on cross-modal semantic mapping, achieving feature-level semantic alignment through semantic reconstruction and loop consistency constraints. This mechanism first introduces a generative alignment model in a unified semantic embedding space, mapping text and image features into a shared semantic domain. It realizes cross-modal mutual translation through inverse generative networks, i.e., generating visual semantic representations from text features and reconstructing text semantics from the generated visual representations, thereby establishing a closed-loop consistency relationship between modalities. This loop-generation structure ensures bidirectional fidelity and reversible consistency of semantic information, effectively reducing the distribution discrepancy of heterogeneous features. Meanwhile, MGLV introduces a semantic-consistency constraint loss to limit the deviation between the generated and original semantic representations, thereby exhibiting stronger robustness against modality heterogeneity and semantic drift, and providing a more stable and interpretable foundation for the subsequent semantic alignment in the fusion stage.

In the fusion stage, the proposed Semantic Confidence Matrix and Modality-Cross Interaction (SCM-MCI) mechanism establishes a dynamic fusion framework driven by confidence-adaptive weighting and bidirectional semantic interaction. It first models the relative importance of different modality features through a semantic confidence matrix. It adaptively regulates feature contributions by normalizing and nonlinearly mapping confidence distributions, thus establishing a semantic-driven dynamic balance among different modalities. Subsequently, the modality-interaction module performs cross-modal semantic association modeling through a bidirectional attention structure, enabling mutual perception and collaborative optimization of text and image features during the fusion process. Through the synergistic mechanism of “confidence-driven and interaction-enhanced”, the model can automatically suppress the interference of low-confidence features under semantic conflict or modality noise scenarios, enhance the discriminative power of dominant modalities, and effectively alleviate modality imbalance and semantic drift problems commonly seen in traditional fusion methods. This mechanism significantly improves the robustness and discriminative consistency of multimodal feature fusion, providing a more interpretable and adaptive fusion strategy for multimodal fake news detection tasks.

Experimental results on multiple multimodal fake news detection datasets demonstrate that the IBKA-MSM framework consistently delivers robust and reliable performance across all major evaluation metrics, including accuracy, Precision, Recall, and F1-score. This confirms the effectiveness and general applicability of the proposed method in areas like semantic alignment, modality fusion, and robust feature modeling. This study achieves synergistic innovation in both algorithm design and semantic modeling, providing a new research paradigm and theoretical support for multimodal fake news detection in complex social media environments.

### 5.2. Future Work

Although the IBKA-MSM framework has achieved remarkable results in feature generation and semantic modeling, there remains considerable potential for further enhancement. Future research can be advanced in the following three directions:

First, the generalization ability of the framework can be verified in more complex multimodal and cross-lingual scenarios. Future research may introduce new modalities, such as audio and video, to explore their semantic consistency modeling and robustness in multi-source heterogeneous environments.

Second, with the rapid development of large language models (LLMs), their semantic priors and knowledge representation abilities can be incorporated into the IBKA-MSM framework to enhance the model’s performance in semantic reasoning, contextual understanding, and cross-modal knowledge alignment, thereby improving the overall semantic fusion depth and interpretability.

Finally, in terms of efficiency and practical application, the framework structure can be further optimized to support real-time detection. Through strategies such as parameter sharing, modality distillation, and hierarchical attention compression, the computational complexity can be significantly reduced while maintaining detection accuracy, improving the model’s deployment performance on large-scale social media platforms.

Overall, the IBKA-MSM framework demonstrates strong innovation and robustness in feature optimization, semantic modeling, and fusion mechanisms. Future research will continue to advance along the directions of efficiency, interpretability, and multimodal generalization, promoting multimodal fake news detection technology toward more intelligent and scalable development.

## Figures and Tables

**Table 1:** Sample Distribution Across Data Source Platforms.

Source	Text Samples	Images
Sina News	2449	1260
Science China	4186	4100
Joint Internet Rumor Refutation Platform	903	902

**Table 2:** Key Hyperparameter Settings in Experiments.

Hyperparameter	Setting
Text embedding dimension	512
Image embedding dimension	512
Number of Transformer layers	2
Number of attention heads	4
Hidden size	128
Learning rate	2 × 10^−4^
Dropout rate	0.2
Focal Loss parameters (α = 1.25, γ = 1.5)	A = 1.25, γ = 1.5

**Table 3:** Multimodal Comparison Experimental Results.

Module	Model	Accuracy (%)	Precision (%)	Recall (%)	F1 (%)
T + I	SAFE	83.79	84.66	87.20	83.61
T + I	EANN	85.28	86.07	81.93	83.31
T + I	MDFEND	91.52	90.42	92.30	91.10
T + I	MVAE	91.52	90.60	93.30	91.23
T + I	MMFakeBuster	92.51	91.46	93.80	92.20
T + I	MCAN	92.76	91.70	93.71	92.41
T + I	IBKA-MSM	**95.80**	**94.02**	**94.27**	**94.14**

**Table 4:** Results of Meta-Heuristic Algorithm Comparison Experiments.

Model	Accuracy (%)	Precision (%)	Recall (%)	F1 (%)
WOA-MSM	66.92	34.10	71.51	46.18
PSO-MSM	96.48	88.83	94.09	91.38
BKA-MSM	97.01	91.58	93.55	92.55
IBKA-MSM	95.80	**94.02**	**94.27**	**94.14**

## Data Availability

The original code and data presented in the study are openly available in GitHub official website at https://github.com/daigege-0107/IBKA-MSM (accessed on 27 October 2025).
